# Trajetorias: a dataset of environmental, epidemiological, and economic indicators for the Brazilian Amazon

**DOI:** 10.1038/s41597-023-01962-1

**Published:** 2023-02-02

**Authors:** Ana C. Rorato, Ana Paula Dal’Asta, Raquel Martins Lana, Ricardo B. N. dos Santos, Maria Isabel S. Escada, Camila M. Vogt, Tatiana Campos Neves, Milton Barbosa, Cecilia S. Andreazzi, Izabel C. dos Reis, Danilo A. Fernandes, Mônica da Silva-Nunes, Anielli R. de Souza, Antonio M. V. Monteiro, Claudia T. Codeço

**Affiliations:** 1 Laboratório de Investigação em Sistemas Socioambientais, Instituto Nacional de Pesquisas Espaciais, São José dos Campos, 12227-900 Brazil; 2 Barcelona Supercomputing Center (BSC), Barcelona, 08034 Spain; 3 Universidade Federal do Pará, Belém, Brazil; 4 Departamento de Ciências Administrativas, Universidade Federal de Santa Maria, Santa Maria, Brazil; 5 Programa de Computação Científica, Fundação Oswaldo Cruz, Rio de Janeiro, 21040-900 Brazil; 6 Laboratório de Ecologia Evolutiva e Biodiversidade, Universidade Federal de Minas Gerais, Belo Horizonte, 31270-901 Brazil; 7 Laboratório de Biologia e Parasitologia de Mamíferos Silvestres Reservatórios, Fundação Oswaldo Cruz, Rio de Janeiro, 21040-900 Brazil; 8 Departamento de Biodiversidad, Ecología y Evolución, Universidad Complutense de Madrid, Madrid, Spain; 9 Laboratório de Imunologia Viral, Instituto Oswaldo Cruz, Fundação Oswaldo Cruz, Rio de Janeiro, 21040-900 Brazil; 10 Departamento de Medicina, Centro de Ciências Biológicas e da Saúde, Universidade Federal de São Carlos, São Carlos, 13565-905 Brazil

**Keywords:** Environmental impact, Malaria, Economics

## Abstract

The Trajetorias dataset is a harmonized set of environmental, epidemiological, and poverty indicators for all municipalities of the Brazilian Legal Amazon (BLA). This dataset is the result of a scientific synthesis research initiative conducted by scientists from several natural and social sciences fields, consolidating multidisciplinary indicators into a coherent dataset for integrated and interdisciplinary studies of the Brazilian Amazon. The dataset allows the investigation of the association between the Amazonian agrarian systems and their impacts on environmental and epidemiological changes, furthermore enhancing the possibilities for understanding, in a more integrated and consistent way, the scenarios that affect the Amazonian biome and its inhabitants.

## Background & Summary

The Amazon biome is under substantial threat due to disputes over its resources by competing rural techno-productive systems associated with the agrarian economy. These disputes have resulted in intense transformations of the forest landscape partially observed by changes in land use and land cover indicators. The agrarian systems associated with large-scale cultivation of temporary crops, extensive livestock and intensive farming, are driving forest cover loss, deforestation, and degradation^1–3^.The increasing pressure on this vast biome that has the highest levels of biodiversity in the world, creates critical socio-environmental conditions that can trigger the emergence of new infectious diseases originating from wild reservoirs^4–6^. Finding social-economic-environmental integrated solutions for the Amazon region demands joint effort and innovation to build collaborative environments for debate and dialogue toward the development of informed multidisciplinary and multicultural processes, knowledge exchange, harmonization of concepts and consolidation of meaningful indicators.

Costa (2009)^7^ defines six rural techno-productive trajectories (*TTs*) in the region. The first three are characterized by production systems based on rural establishments run by “smallholder” or “family-based” arrangements, where economic agents predominantly follow Peasant-type microeconomic reasoning. These production systems are mosaics of permanent and temporary crops (*TT*1), agroforest systems (*TT*2), and small cattle farms with crops (*TT*3). In contrast, the other three production systems are represented by large-scale cattle farming and crop production for animal consumption (*TT*4); large permanent crops, planted forests, and technified silviculture (*TT*5&6); and intensive large-scale temporary crop systems (*TT*7). Their main agents are establishments run by the “agribusiness” or “wage-based” arrangements, where microeconomic decisions are aligned with the large-scale markets and trade of commodities. The Brazilian agrarian census has been the most valuable source of data for the characterization of these agrarian systems, their technologies, productivity, and modes of production^2^.

In Codeço *et al*.^8^, we proposed a framework for the joint debate on the socio-economic, environmental, and health dimensions of municipalities following the above-mentioned TTs. The Trajetorias dataset contains a rich set of indicators that allows for analyzing the spatial and temporal relationship between economic trajectories linked to the agrarian systems’ dynamics, availability of natural resources and disease risk. There are indicators of leishmaniasis, malaria, Chagas disease, and dengue, which are neglected tropical diseases^9^. The prevalences of these infectious diseases are indicative of social and environmental inequalities as the lack of access to proper healthcare and governmental negligence results in increased vulnerability. The environmental indicators are derived from satellite image data and characterize Amazonian municipalities in terms of habitat loss, land use and land cover patterns, transport network density, and climate anomalies. The multidimensional poverty index for rural and urban populations was derived taking into consideration the specificities of the Amazon region.

The dataset was produced by a scientific synthesis research initiative conducted by scientists from natural and social sciences to integrate indicators into a coherent dataset describing the Brazilian Amazon. Its purpose is to contribute to the debate on the future of the Amazon and to be used in investigations of scenarios that impact the biodiversity and human health in the region.

## Methods

### Study area

**Fig. 1:**
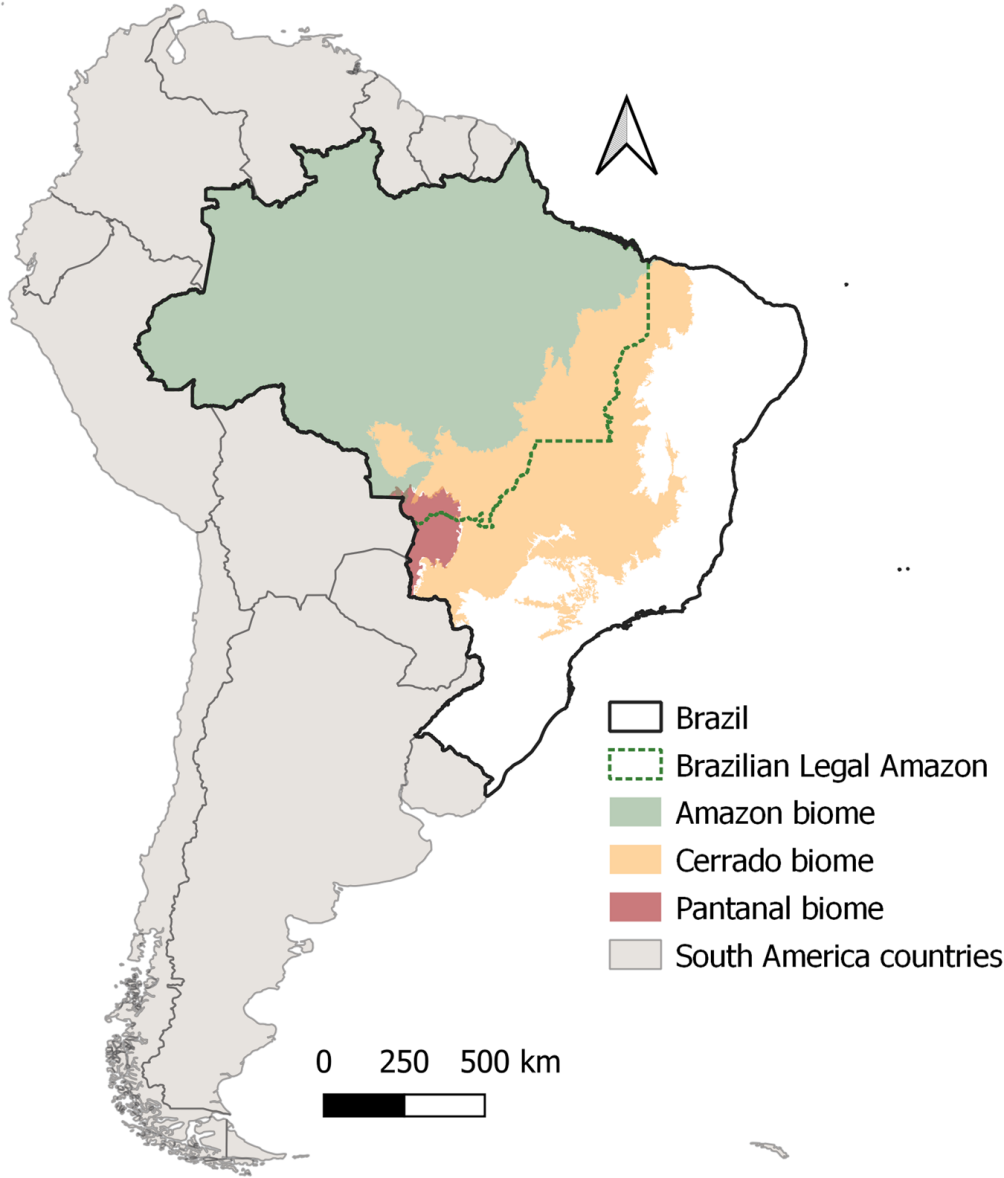
The Brazilian Legal Amazon (BLA) houses the whole Brazilian Amazon biome (predominantly composed of tropical forests), 20% of the Cerrado biome (with a predominance of savannah vegetation) and approximately 40% of the Pantanal biome (the largest continuous wetland in the world), in the state of Mato Grosso.

The Brazilian Legal Amazon (*BLA*) region is a political-administrative area^10^, of about 5 million km^2^, corresponding to approximately 58.9% of the Brazilian territory (Fig. 1). BLA comprises 772 municipalities distributed in 9 states: Acre (22 municipalities), Amapá (16), Amazonas (62), Pará (144), Rondônia (52), Roraima (15), Mato Grosso (141), Tocantins (139), and part of Maranhão (181, of which 21 are only partially included)^11^. Historically, the BLA has faced multiple environmental threats as the occupation of this region advanced. These threats have intensified and receded in response to changes in the strength of the institutions and policies combating deforestation, the control of illicit land market operations^12,13^, the establishment of policies to encourage economic activities such as agriculture and mining^14^, the establishment of infrastructure to favor Agrobusiness Trajectories (such as roads, ports, and hydropower), and the fluctuation in commodities’ prices^15–17^.

In the last 20 years, the Amazon has gone through periods of intense forest loss, with higher deforestation rates in 2004 (27,772 km^2^), followed by a significant reduction between 2005 and 2015 and an increase from 2016 onwards. In the period from 2005 to 2015, the institutions responsible for monitoring and inspecting deforestation were strengthened, with the enaction of policies to combat deforestation, such as the PPCDAm - Action Plan for Prevention and Control of Deforestation in the Legal Amazon, first launched in 2004^18^. Additionally, the implementation of the soy and meat moratorium and the creation of mosaics of Conservation Units, among other policies contributed to the reduction in deforestation. As a result, a significant drop in deforestation rates was observed, with 2012 being the year with the lowest rate (4,571 km^2^) since 1988, the beginning of INPE’s monitoring period. However, this legacy has been heavily impacted by the policies enacted in recent years^19^. After 2016, with changes in Brazil’s environmental policies that decreased governmental investments, social participation, and the dismantling of protective institutions^20–22^, a growing trend in deforestation rates was observed, reaching 13,038 km^2^ in 2021, with the emergence of new fronts of deforestation as in south Amazonas^23,24^, where the largest continuous area of forest is located.

### Study design

**Fig. 2:**
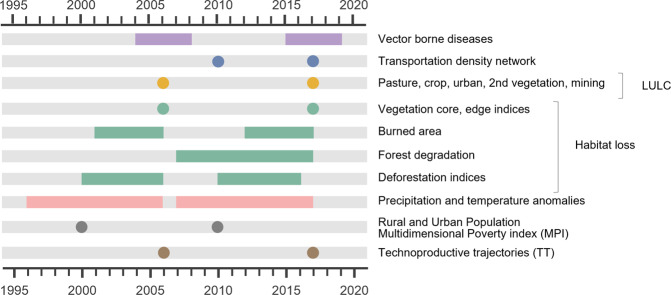
The Trajetorias dataset indicators are computed for two periods, for each municipality, as shown in the diagram. The reference point of each period is the date of agrarian census, from which it is computed the rural techno-productive trajectory. The database harmonizes epidemiological, environmental and economic indicators, as described in the main text.

The Trajetorias dataset contains habitat loss, land use and land cover, human mobility, climate anomalies, the burden of vector-borne diseases, and poverty indices for rural and urban populations for each of the BLA municipalities. These indicators were designed to unveil the specificities of the BLA region, so that the relationships between these dimensions can be explored regarding past and current enacted policies. The first challenge for data harmonization was to define adequate time scales, based on the nature of the data sources and the underlying conceptual model. The Trajetorias dataset relies on four surveys - the two demographic censuses conducted in 2000 and 2010, and the two agrarian censuses conducted in 2006 and 2017, from which were defined fixed timestamps for analysis (Fig. 2). The demographic censuses are the source of data for the multidimensional poverty indices^25^, while the agrarian censuses were the source for the TT characterization^26^. Environmental data come from satellite images collected by several national and international programs, such as the Amazon Deforestation Monitoring Program (PRODES), DEGRAD, and DETER^27^, accounting for changes in landscape that took place between each demographic census and the subsequent agrarian census. Lastly, disease data was obtained from the National Disease Notification System. These data sources were chosen due to their easy accessibility, temporal and spatial coverage, and high quality of data. Environmental indicators and maps were produced using R Core Team 4.2.0 (2022)^28^, ArcGIS 10.4 (ESRI 2016)^29^, Quantum Gis 3.0^30^, and FillCell (Version 2.2.1)^31^, a cell filling script manager developed by INPE. Tables 1–3 summarize all indicators belonging to the environmental, epidemiological and socioeconomic dimensions.

**Table 1:** Description of the environmental indicators in the Trajetorias dataset.

Subdimension	Indicator	Unity	Timeframe	ID
Habitat loss	Deforestation	km^2^/km^2^	[t1, t2] = [2000, 2006] or [2010, 2016]	deorg
Habitat loss	Deforestation	km^2^/km^2^	[t1, t2] = [2000, 2006] or [2010, 2016]	defor
Habitat loss	Forest degradation	km^2^/km^2^	[t1, t2] = [2007, 2017]	dgorg
Habitat loss	Forest degradation	km^2^/km^2^	[t1, t2] = [2007, 2017]	dgfor
Habitat loss	Fire	km^2^/km^2^	[t1, t2] = [2001, 2006] or [2012, 2017]	fire
Habitat loss	Vegetation fragmentation (core)	km^2^/km^2^	t = 2006 or 2017	core
Habitat loss	Vegetation fragmentation (edge)	m/m^2^	t = 2006 or 2017	edge
Land Use and Land Cover	Remnant forest	km^2^/km^2^	t = 2007 or 2017	refor
Land Use and Land Cover	Secondary vegetation	km^2^/km^2^	t = 2006 or 2017	secveg
Land Use and Land Cover	Pasture	km^2^/km^2^	t = 2006 or 2017	pasture
Land Use and Land Cover	Crop	km^2^/km^2^	t = 2006 or 2017	crop
Land Use and Land Cover	Mining	km^2^/km^2^	t = 2006 or 2017	mining
Land Use and Land Cover	Urban area	km^2^/km^2^	t = 2006 or 2017	urban
Transportation networks	Roads network	m/m^2^	t = 2010 or 2017	roads
Transportation networks	Waterways network	m/m^2^	2010	river
Transportation networks	Ports	count	2017	port
Climatic anomalies	Positive precipitation	m^2^/m^2^	[t1, t2] = [1996, 2006] or [2007, 2017]	precp
Climatic anomalies	Negative precipitation	m^2^/m^2^	[t1, t2] = [1996, 2006] or [2007, 2017]	precn
Climatic anomalies	Positive temperature	m^2^/m^2^	[t1, t2] = [1996, 2006] or [2007, 2017]	tempp

**Table 2:** Description of the epidemiological indicators in the Trajetorias dataset.

Subdimension	Indicator	Unity	Timeframe	ID
Vector-borne diseases	Disease	Chagas, CL, VL, Dengue, Falciparum, Vivax, Vivax+Falciparum	[t1, t2] = [2004, 2008] or [2015, 2019]	disease
Vector-borne diseases	Residential zone	rural or urban zones	[t1, t2] = [2004, 2008] or [2015, 2019]	zone
Vector-borne diseases	Disease cases	count	[t1, t2] = [2004, 2008] or [2015, 2019]	cases
Vector-borne diseases	Disease incidence	incidence rate	[t1, t2] = [2004, 2008] or [2015, 2019]	inc

**Table 3:** Description of the socio-economic indicators in the Trajetorias dataset.

Subdimension	Indicator	Unity	Timeframe	ID
MPI	Multidimensional Poverty Incidence (H)	%	t = 2000 or 2010	h
MPI	Multidimensional Poverty Intensity (A)	%	t = 2000 or 2010	A
MPI	Deprivations	dimensionless	t = 2000 or 2010	carpon
MPI	Multidimensional Poverty Index	MPI = A*H	t = 2000 or 2010	mpi
MPI	Contribution of the Health Dimension	%	t = 2000 or 2010	csaude
MPI	Contribution of the Education Dimension	%	t = 2000 or 2010	ceduca
MPI	Contribution of habitation and sanitation	%	t = 2000 or 2010	ccv
MPI	Contribution of work and private consumer goods	%	t = 2000 or 2010	cnv
MPI	People	count	t = 2000 or 2010	totpescar
Population	Proportion of urban population in 2000	%	t = 2000	prop_urb2000
Population	Proportion of urban population in 2010	%	t = 2010	prop_urb2010
Population	Proportion or rural population in 2000	%	t = 2000	prop_rur2000
Population	Proportion or rural population in 2010	%	t = 2010	prop_rur2010

### Environmental dimension

#### Habitat loss

The indicators describing habitat loss include deforestation, forest degradation, burned area and vegetation fragmentation. Deforestation indicators were calculated using the data from PRODES, a Program that monitors clear-cutting deforestation (complete suppression of the forest) in areas of forest physiognomy in the Legal Amazon since 1988^32^. Two indicators were calculated for the 7-year periods that preceded the 2006 and 2017 agrarian censuses: *deorg* is the ratio between the total deforested area during the period and the original forest area, as recorded in 1988; while *defor* is the ratio between the total deforested area during the period and the total forested area present at the beginning of each period (2000–2006 and 2010–2016):

1 $$deorg(m,{t}_{1},{t}_{2})=\frac{{\rm{m}}\text{'}{\rm{s}}\;{\rm{deforested}}\;{\rm{area}}\;{\rm{between}}\;{t}_{1},{t}_{2}}{{\rm{m}}\text{'}{\rm{s}}\;{\rm{original}}\;{\rm{forest}}\,{\rm{area}}}$$

2 $$defor(m,{t}_{1},{t}_{2})=\frac{{\rm{m}}\text{'}{\rm{s}}\,{\rm{deforested}}\;{\rm{area}}\,{\rm{between}}\,{t}_{1},{t}_{2}}{{\rm{m}}\text{'}{\rm{s}}\,{\rm{forest}}\,{\rm{area}}\,{\rm{in}}\,{t}_{1}}$$

where [*t*_1_, *t*_2_]=[2000,2006] or [2010,2016] and *m* is the municipality.

As so, *deorg* and *defor* provide measures of forest cover change relative to original and recent forest, respectively. These two indicators can be used to distinguish municipalities with different dynamics of deforestation between the periods of study.

Forest degradation, differently from clear-cut deforestation, is the process of gradual loss of forest cover due to selective logging and/or forest fires, considering a minimum mapping area of 6.25 ha^27^. Forest degradation data come from the DEGRAD program, ran by INPE between 2007 and 2016^27^, and the Real Time Deforestation Detection System (DETER) from 2016 on^33,34^. Data from the two sources were merged and the indicators were calculated as follows: *dgorg* is the ratio between the degraded area where forest degradation was detected at least once in the municipality between 2007 and 2017 divided by the original forest area, as measured in 1988; while *dgfor* is the ratio between the same degraded area and the forest area present in 2007 in the municipality. Note that *dgorg* and *dgfor* observation period is different from other deforestation indicators due to restrictions in data availability. Therefore, for these two indicators, there is only one measurement per municipality.

3 $$dgorg(m,{t}_{1},{t}_{2})=\frac{{\rm{m}}\text{'}{\rm{s}}\;{\rm{area}}\,{\rm{with}}\;{\rm{detected}}\;{\rm{forest}}\;{\rm{degradation}}\;{\rm{between}}\,{t}_{1},{t}_{2}}{{\rm{m}}\text{'}{\rm{s}}\,{\rm{original}}\;{\rm{forested}}\;{\rm{area}}}$$

4 $$dg\,for(m,{t}_{1},{t}_{2})=\frac{{\rm{m}}\text{'}{\rm{s}}\;{\rm{area}}\;{\rm{with}}\;{\rm{detected}}\;{\rm{forest}}\;{\rm{degradation}}\;{\rm{between}}\;{t}_{1},{t}_{2}}{{\rm{m}}\text{'}{\rm{s}}\;{\rm{forested}}\;{\rm{area}}\;{\rm{in}}\;{t}_{1}}$$

where [*t*_1_, *t*_2_] = [2007,2017] and *m* is the municipality.

Forest fire is an important cause of biodiversity loss, carbon emission, and land cover conversion^35^. The indicator *fire* computes the proportion of the total municipality area that was burned in the two periods 2001–2006 and 2012–2017. To create these indicators, we used the MODIS’ (NASA’s Moderate Resolution Imaging Spectroradiometer) Global Burned Area Product (Collection 6) which detects burned areas daily at a spatial resolution of 500 m^36^. Note that this indicator disregard multiple fires in the same area.

5 $$fire(m,{t}_{1},{t}_{2})=\frac{{\rm{m}}\text{'}{\rm{s}}\;{\rm{area}}\;{\rm{burned}}\;{\rm{at}}\;{\rm{least}}\;{\rm{once}}\;{\rm{between}}\;{t}_{1},{t}_{2}}{{\rm{m}}\text{'}{\rm{s}}\;{\rm{area}}}$$

where [*t*_1_, *t*_2_]=[2001,2006] or [2012,2017] and *m* is the municipality.

An unequal rate of vegetation loss creates a spatial fragmentation of the landscape, potentially amplifying deleterious effects on biodiversity and ecosystem functioning^37^. For example, new forest edges and smaller fragments increase tree mortality, fire frequency, amplify vector-borne diseases, and sylvatic host populations^38^. Vegetation fragmentation in the Trajetorias dataset is measured using two indicators, calculated from 2006 and 2017 yearly land use and land cover (LULC) maps, with 250 meters spatial resolution. These maps were produced by Camara *et al*.^39^ using image time series from MOD13Q1 product, from MODIS (collection 6). We combined all-natural vegetation classes (forest and savanna) into a single’natural vegetation’ class. The indicator *core* measures the proportion of the municipality’s total area covered by natural vegetation core areas in 2006 and 2017. A core is defined as the area within a patch separated from the border by a predefined distance^40^. In this case, we defined 300 m, where tree mortality and reduced growth is high due to the edge effect^41^. The edge effect (*edge*) was calculated as the ratio between the total length of the edges of the vegetation fragments (in meters) and the square root of the total vegetation area per municipality, in 2006 and 2017. The two indicators were calculated using the spatialEco package in R^42^.

6 $$core(m,t)=\frac{{\rm{m}}\text{'}{\rm{s}}\;{\rm{core}}\;{\rm{area}}\;{\rm{at}}\;t}{{\rm{m}}\text{'}{\rm{s}}\;{\rm{area}}}$$

where *t* = 2006 or 2017 and *m* is the municipality.

7 $$edge(m,t)=\frac{{\rm{total}}\;{\rm{edge}}\;{\rm{length}}\;{\rm{in}}\;{\rm{m,}}\;{\rm{at}}\;t}{\sqrt{{\rm{m}}\text{'}{\rm{s}}\;{\rm{vegetation}}\;{\rm{area}}\;{\rm{at}}\;t}}$$

where *t* = 2006 or 2017.

#### Land use and land cover

**Fig. 3:**
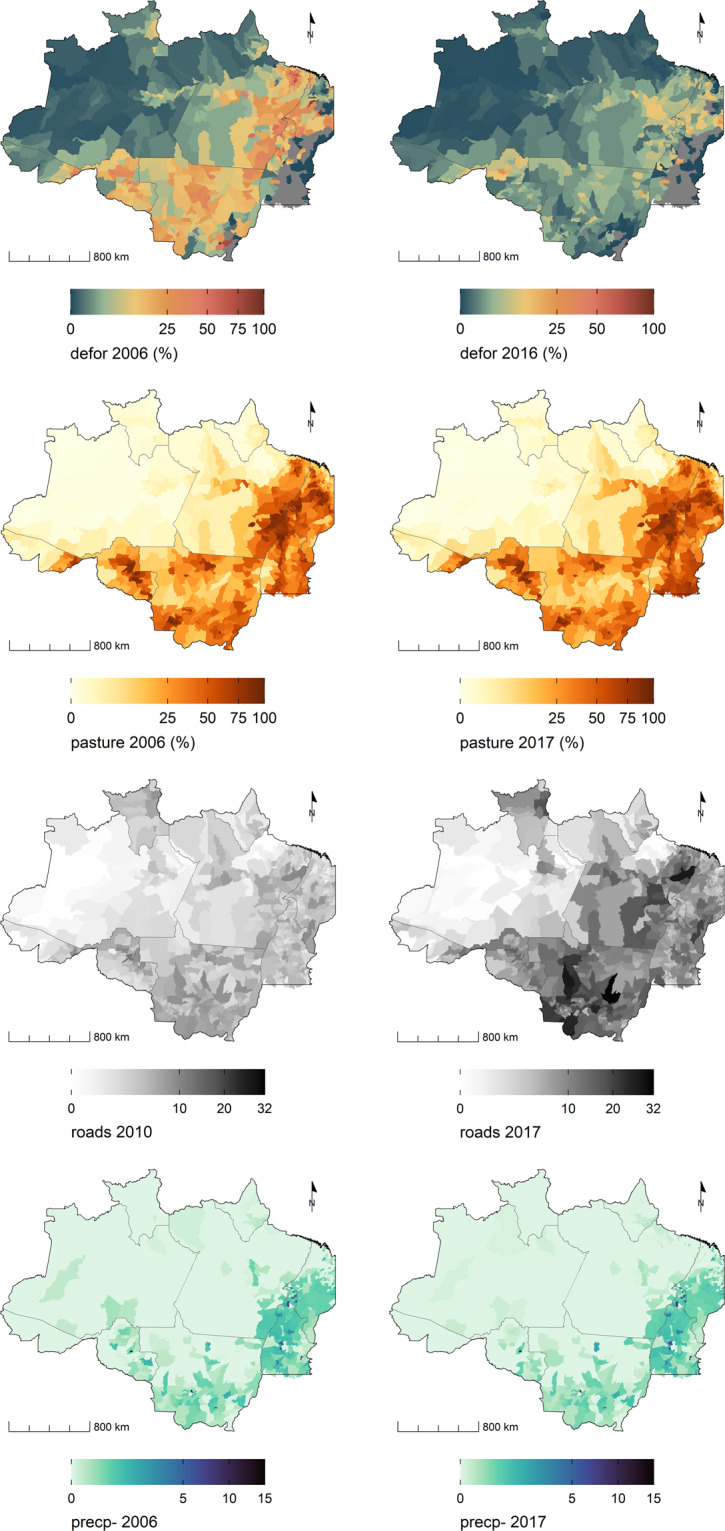
A subset of environmental indicators from the Trajetorias dataset allows the comparison of the rate of deforestation, and the expansion of pasture between 2006 and 2016/2017, the expansion of the road network, and the distribution of negative precipitation anomalies in the Brazilian Legal Amazon region.

Changes in land use and land cover in tropical forests are indicators of important environmental impacts. In the Amazon region, the general transition pattern starts with forest being removed from the land that will be used for agricultural or livestock production. Later, the cultivation or pasture is abandoned and secondary forest grows over the region^43^. Satellite images show transitions between different land use and land cover classes that can be traced at short- and long-term timescales (Fig. 3). These landscape footprints are later used to characterize environmental trajectories^8,44,45^. The following indices describe the landscape of each municipality in 2006 and 2017:

*refor* is the proportion of remnant forest area in 2007 and 2017 in relation to the municipality’s original forest area as recorded in 1988, estimated using forest data from PRODES; *secveg* is the proportion of the municipality’s area covered with secondary vegetation in 2006 and 2017, computed using data from the annual LULC maps classified by Camara *et al*.^39^.

8 $$re\;for(m,t)=\frac{{\rm{m}}\text{'}{\rm{s}}\;{\rm{area}}\;{\rm{with}}\;{\rm{remnant}}\;{\rm{forest}}\;{\rm{at}}\;t}{{\rm{m}}\text{'}{\rm{s}}\;{\rm{original}}\;{\rm{forested}}\;{\rm{area}}}$$

where *t* = 2007 or 2017 and *m* is the municipality.

9 $$secveg(m,t)=\frac{{\rm{m}}\text{'}{\rm{s}}\;{\rm{area}}\;{\rm{with}}\;{\rm{secondary}}\;{\rm{vegetation}}\;{\rm{at}}\;t}{{\rm{m}}\text{'}{\rm{s}}\;{\rm{area}}}$$

where *t* = 2006 or 2017.

Estimates of agricultural land size were computed as the proportion of the municipality’s area classified as pasture (*pasture*) or crop (*crop*) in 2006 and 2017 using the above-mentioned LULC maps from the MODIS time series^39^.

10 $$pasture(m,t)=\frac{{\rm{m}}\text{'}{\rm{s}}\;{\rm{area}}\;{\rm{with}}\;{\rm{pasture}}\;{\rm{at}}\;t}{{\rm{m}}\text{'}{\rm{s}}\;{\rm{area}}}$$

where *t* = 2006 or 2017.

11 $$crop(m,t)=\frac{{\rm{m}}\text{'}{\rm{s}}\;{\rm{area}}\;{\rm{with}}\;{\rm{crop}}\;{\rm{at}}\;t}{{\rm{m}}\text{'}{\rm{s}}\;{\rm{area}}}$$

where *t* = 2006 or 2017.

The proportion of the municipality’s area classified as urban land (*urban*) in 2006 and 2017 was estimated using LULC maps available at the Mapbiomas Project, Collection 6^46^.

12 $$urban(m,t)=\frac{{\rm{m}}\text{'}{\rm{s}}\;{\rm{area}}\;{\rm{classified}}\;{\rm{as}}\;{\rm{urban}}\;{\rm{at}}\;t}{{\rm{m}}\text{'}{\rm{s}}\;{\rm{area}}}$$

where *t* = 2006 or 2017.

Mining activity was estimated as the proportion of the municipality’s area with mining activities (*mining*), both industrial and artisanal, in 2006 and 2017, using annual LULC maps from the Mapbiomas Project (Collection 6)^47^. The mapping of the mining class in Collection 6 of MapBiomas uses Deep Learning and reference data from CPRM (Brazilian Geological Service), Ahk Brasilien (Chamber of Commerce and Industry), INPE, and ISA (Socio-environmental Institute), which encompasses activities of garimpo (artisanal mining in Portuguese) and industrial mining.

13 $$mining(m,t)=\frac{{\rm{m}}\text{'}{\rm{s}}\;{\rm{area}}\;{\rm{with}}\;{\rm{mining}}\;{\rm{activities}}\;{\rm{at}}\;t}{{\rm{m}}\text{'}{\rm{s}}\;{\rm{area}}}$$

where *t* = 2006 or 2017.

#### The density of transportation networks

Human occupation of the Amazon region is concentrated along road and river transportation networks^15,16,48^, and denser road or river networks are proxies for higher human mobility and connectivity. The introduction of dengue^49^ and COVID-19^50^ in the Amazon region are associated with road networks while outbreaks of malaria are linked to new human settlements alongside roads^51,52^. The Trajetorias dataset has a road density indicator (*roads*) computed as the total length of roads in the municipality divided by the square root of the total municipality’s area, in 2010 and 2017 (Fig. 3).

14 $$roads(m,t)=\frac{{\rm{sum}}\;{\rm{of}}\;{\rm{all}}\;{\rm{road}}\;{\rm{lengths}}\;{\rm{in}}\;{\rm{m,}}\;{\rm{at}}\;t}{\sqrt{{\rm{m}}\text{'}{\rm{s}}\;{\rm{area}}}}$$

where *t* = 2010 or 2017 and *m* is the municipality.

The 2010 road data was obtained from the LAPIG (Laboratory of Image Processing and Geoprocessing at the Federal University of Goiás) map platform that integrates data from IBGE (Brazilian Institute of Geography and Statistics), DNIT (National Infrastructure and Transport Department), and ANTT (National Land Transportation Agency)^53^. The 2017 road networks data was obtained from the RAISG (Amazon Network of Georeferenced Social and Environmental Information)^54^, which is based on data collected by IBGE.

The fluvial network density was computed as the total length of waterways (including all waterways, regardless of level of traffic) within the municipality divided by the square root of the municipality’s area. Data from 2010 was obtained from the National Department of Transport Infrastructure (DNIT) and the National Water Transport Agency (ANTAQ). An indicator of mobility infrastructure is the total number of ports per municipality, obtained from the DNIT 2017.

15 $$river(m)=\frac{{\rm{sum}}\;{\rm{of}}\;{\rm{all}}\;{\rm{waterway}}\;{\rm{lengths}}\;{\rm{in}}\;m}{\sqrt{{\rm{m}}\text{'}{\rm{s}}\;{\rm{area}}}}$$

16 $$port(m)={\rm{number}}\;{\rm{of}}\;{\rm{ports}}\;{\rm{in}}\;{\rm{m}}$$

where *m* is the municipality.

#### Climatic anomalies

The Amazon region is at great risk for climate change due to synergistic effects between global warming and local landscape change, driven by deforestation, forest fires, and forest fragmentation^55^. Precipitation anomalies are expected to cause large health and ecological impact in a region strongly dependent on its rainy season. Extreme drought events can increase the risk of forest fires, disrupt fluvial transportation, and reduce water quality; while extreme precipitation can lead to floods, also affecting vector-borne diseases spread, mobility, among other effects^56^. The Trajetorias dataset contains indicators that measure the spatial magnitude of climatic anomaly events in each municipality. Extreme variations from the climatological normal constitute a hazard, affecting local livelihoods and increasing the risk of diseases and disasters in the Amazon region.

Minimum temperature (*temp*) and precipitation (*prec*) anomaly indicators are estimated during two time intervals, from 1996 to 2006 and from 2007 to 2017. The data source is the Historical Monthly Weather data from the WorldClim database (CRU-TS 4.03^57^ downscaled with WorldClim 2.1^58^) which provides access to global monthly weather data with a spatial resolution of 21 km^2^. Climatic anomalies are calculated from the variation of a measure in relation to the climatological normal of that same variable. The climatological normal was calculated from the 30-year climatological average^59–62^ of each municipality using time series from 1961 to 1990. This period is used by the National Institute of Meteorology (INMET) to compute climatological baselines in Brazil. In the Amazon region, it also represents a period in which disturbances in the forest cover were less intense (1961–1990).

Negative and positive precipitation anomalies were measured during the dry season to detect extremely dry years. In general, changes in precipitation patterns tend to affect the reproduction, development and population dynamics of vector arthropods. In particular, drought events tend to concentrate mosquitoes in small pools of water (or water storage containers in urban areas), reducing the presence of competitors and predators and accumulating eggs that are resistant to drought, leading to subsequent increases in mosquito numbers and disease outbreaks when the rainy period returns^63–66^. Negative and positive minimum temperature anomalies were measured during the cooler season. The minimum temperature is a critical factor in many regions for the survival, development rates, and biting rates of the mosquito that transmits dengue^67^. Although the region’s temperature is warm, friagens (cool temperatures) occur due to gusts of polar masses.

After calculating the climatological average for the municipalities, we applied a Spatial Kluster Analysis by Tree Edge Removal (SKATER)^68^ to classify the Amazonian municipalities into distinct climatological regions and for each, we were able to identify the three coolest and driest months. Only data from these months were used in the indicators, respectively. Figure S1 in Supplementary Information (SI) shows the location of the five regions and their time series of air minimum temperature from 1961 to 2017. A trend of increasing temperature is evident, being more pronounced on the eastern Amazon.

Anomaly indicators were computed as follows: 1) for each pixel of c.a. 21 km^2^, we estimated the average (mean) and standard deviation (sd) of *prec* and *temp* using the 30-year time series; 2) then, a month was considered anomalous if *prec* > mean(*prec*) + 1.5*sd(*prec*) or *prec* <mean(*prec*) - 1.5*sd(*prec*), indicating a positive or negative precipitation anomaly, respectively. Likewise, we computed the indicators for positive and negative minimum temperature anomalies. Thus, for each municipality, we counted all anomalous pixels in the two periods of study: 1996 to 2006 and 2007 to 2017. In each period, anomaly events were counted for 33 months (11 years × 3 months per year, corresponding to the drier/cooler months). Finally, the indicators of precipitation and minimum temperature anomalies were computed using the following equations:

17 $$precp(m,{t}_{1},{t}_{2})=\frac{{\rm{sum}}\;{\rm{of}}\;{\rm{areas}}\;{\rm{with}}\;{\rm{positive}}\;{\rm{precipitation}}\;{\rm{anomalies,}}\;{\rm{in}}\;{\rm{m,}}\;{\rm{monthly,}}\;{\rm{between}}\;{t}_{1},{t}_{2}}{N\times {\rm{m}}\text{'}{\rm{s}}\;{\rm{area}}}$$

18 $$precn(m,{t}_{1},{t}_{2})=\frac{{\rm{sum}}\;{\rm{of}}\;{\rm{areas}}\;{\rm{with}}\;{\rm{negative}}\;{\rm{precipitation}}\;{\rm{anomalies,}}\;{\rm{in}}\;{\rm{m,}}\;{\rm{monthly,}}\;{\rm{between}}\;{t}_{1},{t}_{2}}{N\times {\rm{m}}\text{'}{\rm{s}}\;{\rm{area}}}$$

19 $$tempp(m,{t}_{1},{t}_{2})=\frac{{\rm{sum}}\;{\rm{of}}\;{\rm{areas}}\;{\rm{with}}\;{\rm{positive}}\;{\rm{temperature}}\;{\rm{anomalies,}}\;{\rm{in}}\;{\rm{m,}}\;{\rm{monthly,}}\;{\rm{between}}\;{t}_{{\rm{1}}}{\rm{,}}{t}_{{\rm{2}}}}{N\times {\rm{m}}\text{'}{\rm{s}}\;{\rm{area}}}$$

where *N* = 33 months analyzed, *m* is the municipality, and [*t*_1_, *t*_2_] = [1996,2006] or [2007,2017]. For minimum temperature, we did not find any negative anomaly, that is, a month with minimum temperature below the climatology-defined threshold. This can be attributed to the trend of increasing temperatures observed in the last decades.

A subset of environmental indicators is presented in Fig. 3. Figures S2 and S3 in SI show the correlation between the indicators of the environmental dimension.

### Epidemiological dimension

**Fig. 4:**
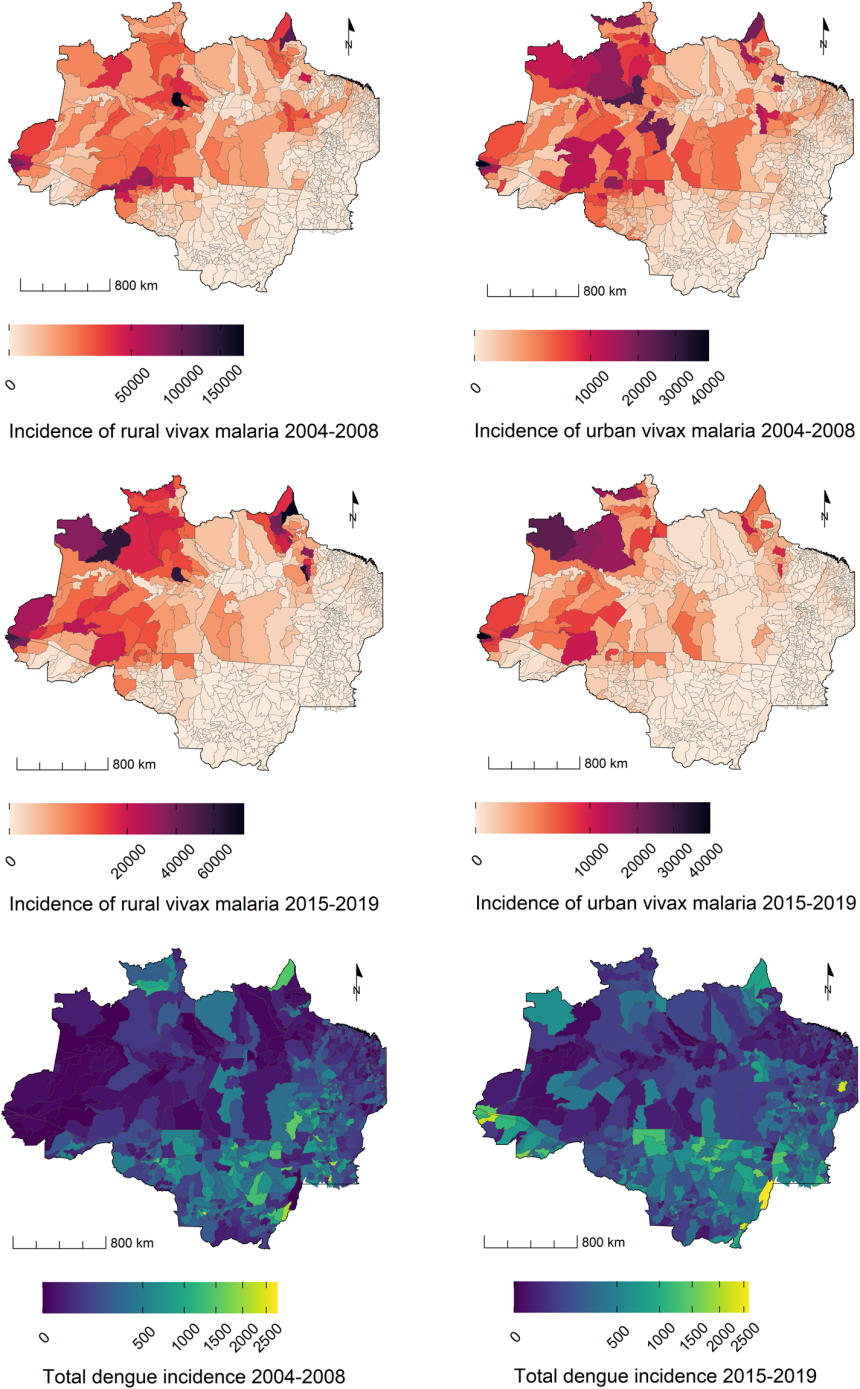
A subset of epidemiological indicators from the Trajetorias dataset showing the incidence rate of malaria by *P. vivax* and *P. falciparum*, and dengue in the Brazilian Legal Amazon region.

The risk of vector-borne diseases in the Amazon region is expected to change as a results of climate and environmental changes^69^. Different economic activities associated with competing technoproductive rural trajectories are also expected to impact the social-ecological landscape with direct effects on the emergence and reemergence of transmissible diseases^8^. The Trajetorias dataset contains total counts and incidence rates of Chagas disease, visceral leishmaniasis, cutaneous leishmaniasis, malaria and dengue per municipality, in the 2004–2008 and 2015–2019 periods. These two periods are centered on the date of the Brazilian agrarian censuses. For all diseases but dengue, the indicators are further stratified by rural and urban zone. The incidence rate is a measure of disease frequency for dynamic populations expressed as the number of cases per person in a year^70^. It was estimated dividing the number of cases by the population in the middle of the period multiplied by the number of years of observation (5 years) (Fig. 4).

20 $$cases(d,m,z,{t}_{1},{t}_{2})={\rm{sum}}\;{\rm{of}}\;{\rm{cases}}\;{\rm{of}}\;{\rm{the}}\;{\rm{disease}}\;{\rm{d,}}\;{\rm{in}}\;{\rm{zone}}\;{\rm{z}}\;{\rm{of}}\;{\rm{m,}}\;{\rm{between}}\;{t}_{1},{t}_{2}$$

21 $$inc\left(d,m,z,{t}_{1},{t}_{2}\right)=\frac{cases\left(d,m,z,{t}_{1},{t}_{2}\right)}{pop\left(m,z,{t}_{({t}_{2}-{t}_{2}/2)}\right)\times 5\;{\rm{years}}}\times 1{0}^{5}$$

where [*t*_1_, *t*_2_] = [2004,2008] or [2015,2019], *d* is the disease (chagas, VL (visceral leishmaniasis), CL (cutaneous leishmaniasis), Vivax (malaria by *Plasmodium vivax*), Falciparum (malaria by *P. falciparum*), Vivax + Falciparum (mixed malaria infection)), *m* is the municipality and, *z* is the zone of residence (rural or urban).

The estimation of the population in the denominator is described in the next section. The data source for the leishmaniasis, Chagas, and dengue is the official disease notification system (SINAN), accessed at the public portal DataSUS Tabnet (website1). For malaria, the source is the SIVEP-malaria database.

To aid the interpretation of the epidemiological indicators, Table S1 in the SI provides more information on the occurrence and location of epidemics during the two periods. Figure S4 shows the correlation between the indicators of the epidemiological dimension.

### Socio-economic dimension

#### Techo-productive trajectories

The dominant technological trajectories (TTs) of each municipality, as measured in 2006 and 2017, by Costa (2021)^2^ and (2022)^26^, are found in^71^.

#### Rural and urban population counts

The Trajetorias dataset contains population counts that can be used for the estimation of epidemiological indicators, such as disease incidence rates. Total urban and rural population counts per municipality are available from the 2000 and 2010 censuses. For the inter-census period, only the estimates of population totals are available, and are not divided in rural and urban counts. These counts were estimated using the proportion of rural and urban population in each municipality captured in the 2010 census.

#### Multidimensional poverty index

Measuring poverty in the Amazonian region is a complex task as it requires an understanding of issues that transcend standard economic indicators. The approach here was to measure the degree of family deprivation in rural and urban areas, which can affect and be affected by environmental and economic changes (5). As so, the Trajetorias dataset contains a rural and a urban Multidimensional Poverty Index (MPI) developed specifically for the region.

Following the method proposed by Alkire and Foster (2011)^72^, the MPI-Trajetorias was defined with three dimensions: Health, Education, and Living Conditions. The Living Conditions dimension was further divided into two sub-dimensions: (1) housing and collective services; and (2) employment and private consumption goods. Indicators for each dimension were chosen from the 2000 and 2010 census databases^25^ by a broad and interdisciplinary group of experts, including epidemiologists, economists, ecologists, biologists, geographers, medical doctors, and social scientists. For 2000, 16 indicators were selected for the rural MPI and 15 for the urban MPI. For 2010, the number of indicators was greater due to changes in the Census questionnaire: 19 indicators for rural MPI and 18 for urban MPI (Table 3). Each household in the census was initially identified as rural and urban and received a score for each indicator where 1 indicated a situation of deprivation, and 0 otherwise. Multidimension poverty means simultaneous deprivation in multiple dimensions. To compute it, a weighted deprivation score is computed where each dimension (Health, Education, and two classes of Living Conditions) receives a equal weight 1/4. Within each dimension, all indicators are equally weighted (Table S1). This method is described in^73^.

A households is considered multidimensionally poor if its deprivation score exceeds a threshold, defined here as 0.25. The MPI at municipal level is the combination of two components: (i) the incidence component (H) that measures the proportion of households in that population living in a multidimensional poverty condition; and (ii) the intensity component (A) which is a measure of the average household deprivation score^74^.

22 $$MPI(m,t)=H\times A$$

where:

23 $$H(m,t)=\frac{{\rm{number}}\;{\rm{of}}\;{\rm{multi}}\;{\rm{dimensionally}}\;{\rm{poor}}\;{\rm{households}}\;{\rm{in}}\;{\rm{m,}}\;{\rm{at}}\;{\rm{t}}}{{\rm{households}}\;{\rm{in}}\;{\rm{m,}}\;{\rm{at}}\;{\rm{t}}}$$

24 $$A(m,t)=\frac{{\rm{sum}}\;{\rm{of}}\;{\rm{household}}\text{'}{\rm{s}}\;{\rm{deprivations}}\;{\rm{in}}\;{\rm{m,}}\;{\rm{at}}\;{\rm{t}}}{{\rm{households}}\;{\rm{in}}\;{\rm{m,}}\;{\rm{at}}\;{\rm{t}}}$$

where *m* is the municipality and *t* = 2000,2010.

25 $$ContribDim(m,t,j)=\frac{{\rm{sum}}\;{\rm{of}}\;{\rm{deprivations}}\;{\rm{at}}\;{\rm{dimension}}\;{\rm{j}}\;{\rm{in}}\;{\rm{m,}}\;{\rm{at}}\;{\rm{t}}}{{\rm{MPI}}\;{\rm{in}}\;{\rm{m,}}\;{\rm{at}}\;{\rm{t}}}$$

where *j* are the four MPI dimensions.

All these indicators are found in the dataset. In addition, the user will find the average household deprivation score per municipality and the contribution of each dimension to the MPI Index. The variables and weights used to calculate the rural and urban multidimensional poverty indices are detailed in Table S2. The correlation between the indicators of the economic dimension is shown in Figure S5.

## Data Records

The dataset is organized as a set of.csv tables linked by a geographic id (7-digit municipality code, 2-digit state code), that is linkable to publicly available shapefiles and other harmonized datasets. All indicators, metadata tables and data sources referring to the Trajetorias dataset are deposited in the Zenodo repository^75^.

## Technical Validation

All indicators were computed using the 2017 IBGE municipal grid^25^. As the Brazilian territorial division was altered by changes in the municipalities over time, we harmonize the grids by observing the evolution of the municipal territorial division available in IBGE^76^. In the period from 2000 to 2017, 16 municipalities were established in the BLA (Bom Jesus do Araguaia, Colniza, Conquista d’Oeste, Curvelândia, Ipiranga do Norte, Itanhangá, Nova Nazaré, Nova Santa Helena, Novo Santo Antônio, Rondolândia, Santa Cruz do Xingu, Santa Rita do Trivelato, Santo Antônio do Leste, Serra Nova Dourada, Vale de São Domingos e Mojuí dos Campos). As a new municipality can be the result of the merger of fractions of several other municipalities, we used the 2017 municipal grid as a reference because it is the most recent and with more accurate spatial data. For the period prior to their establishment, these municipalities were assigned the indicators of the municipalities of origin.

The data on clear-cut deforestation from PRODES, generated annually since 1988 by INPE, is a consolidated dataset used as a reference for monitoring forest loss in the Amazon region, and it is officially used for public policies purposes and academic studies. According to Maurano *et al*.^77^, the PRODES deforestation data for 2014 showed 93% overall accuracy level, with omission and inclusion rates estimated at 7% and 1.5%, respectively. There were missing data due to the presence of clouds and in the municipalities cut by the 44° meridian in Maranhão. To minimize issues due to the presence of clouds: we chose periods in which cloud coverage was lower and excluded the period from 1997 to 2000, in which some municipalities were not mapped in Maranhão, Pará, and Amapá. For the municipalities that were cut by the 44° meridian in Maranhão, we calculated the percentage of deforestation in relation to the original forest area within the observed area in the municipality. The forest degradation indicator was calculated using a combination of DEGRAD multitemporal data from 2007 to 2016 that were obtained with Landsat images at 30 m resolution and DETER data for the year 2017, whose sensors have a spatial resolution of 56 to 64 m. However, there may be an underestimation of the degradation in 2017 concerning DEGRAD, mainly related to polygons of small size. Regarding the land use and land cover maps produced by Camara *et al*. 2020, the quality assessment using k-fold cross-validation of the training samples indicates an overall accuracy of 99.22%.

The disease database, SINAN, suffered some changes during the study period that affected the definition of cases and the codification of some variables. To guarantee consistency in the harmonization process, we consulted the original forms and official documentation. We also compared our dataset with bulletins produced by the Ministry of Health. The allocation of cases to rural and urban areas by the local health authorities has uncertainties that can affect incidence estimations. The definition of rural and urban households can be arbitrary in the region, where a gradient of rurality is observed^78^. These details should be taken into consideration when using the data. Moreover, the quality of disease diagnosis may vary. Malaria and Chagas identification are more precise since they are based on specific microscopic examinations. On the other hand, dengue diagnosis relies mostly in clinical symptoms. Therefore, the number of identified cases may underestimate the amount of real cases. Since the public health system in Brazil is comprehensive and has high spatial coverage, we assume that the notification effort was homogeneous during the study period.

The Demographic Census is the most complete source of populational data in Brazil. Since the questionnaires changed from 2000 to 2010 censuses, some variables are defined in slightly different ways in these two years and researchers should use caution when making comparisons and trend interpretations. The MPI Index is not be comparable between 2000 and 2010, as they use some different variables. One aspect to be noted is the weight that the rural environment provides in the composition of the general indicator of the MPI-Trajetorias. In 2000 the general MPI-Trajetorias Index was 0.41, standing out the states of Maranhão, Amazonas and Pará which had the highest average MPI and are also the most populous states in the Legal Amazon, influencing the composition of the indicator. In all states, the rural MPI, on average, was higher than the urban one. This aspect occurs due to known limitations in the demographic census sample questionnaire that does not adequately capture important aspects of the rural Amazon. For example, particularly in the Amazon, there is a lack of questions on the quality of life and consumption practices associated with rural systems operated by peasant agents^3,79^. These first results bring evidence of the need to adapt the demographic census sample questionnaire variables for the rural Amazonian context.

## Usage Notes

**Fig. 5:**
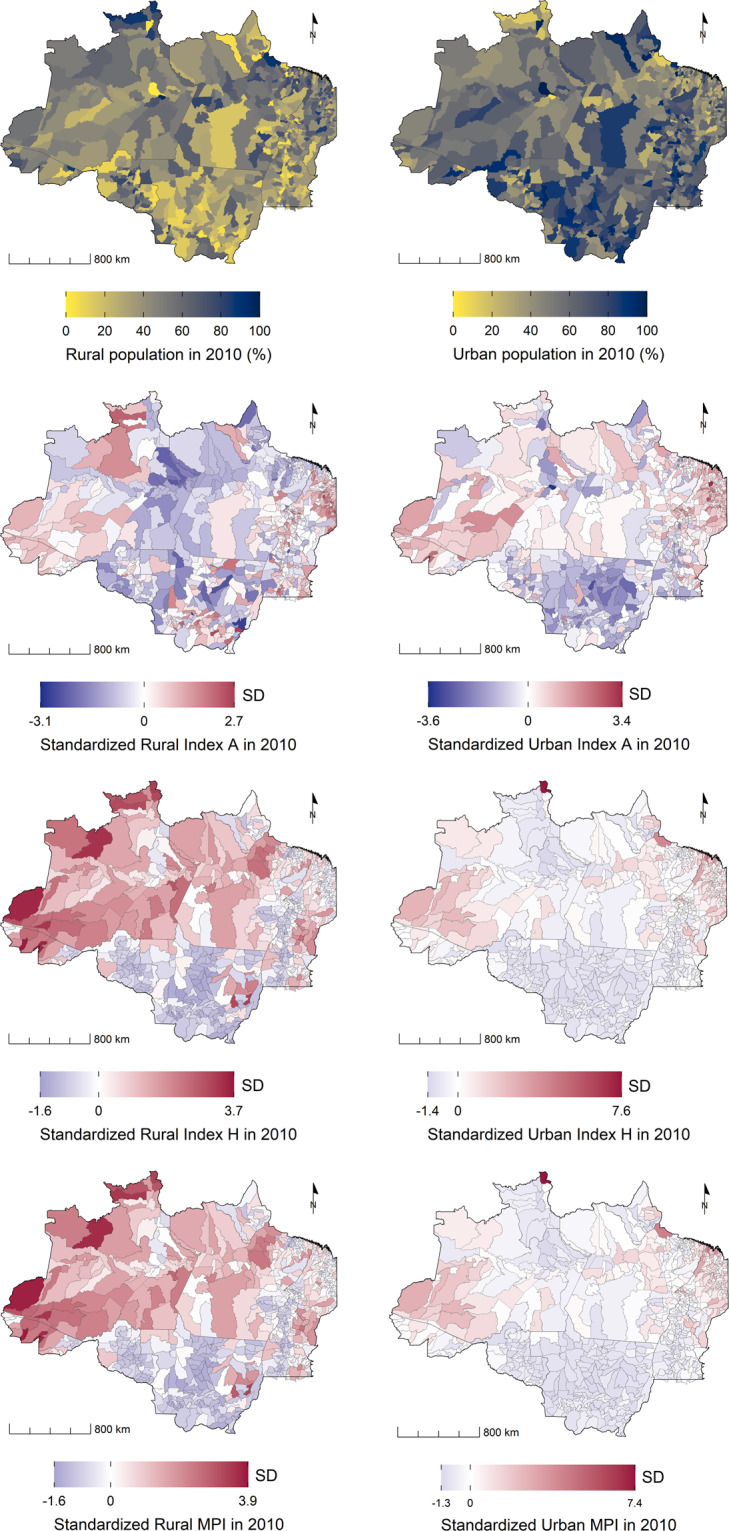
A subset of socio-economic indicators from the Trajetorias dataset showing the distribution of the rural and urban population in the Brazilian Legal Amazon region, 2010. The standardized indices show the deviation of the poverty indices in rural and urban populations, in relation to the overall mean of the region. A = multidimension poverty intensity; H = multidimension poverty incidence, MPI = AxH = multidimension poverty index.

The Trajetorias indicators can be used to assess environmental changes in the BLA during the first two decades of the *XXI*^*th*^ century, and its association with vector borne diseases, poverty, and rural economy. The dataset can be easily integrated with other databases from Brazil, at the municipal level^80^. For future studies, we recommend harmonizing the Trajectories data set with annual environmental data provided by PRODES and DETER, both programs developed by INPE, and which can be accessed on the Terrabrasilis platform (http://terrabrasilis.dpi.inpe.br/). Possible applications include assessing changes in chronic diseases, as well as air and water quality. Figures 3 to 5 exemplify visualizations that can be derived from this dataset. A complete collection of ready-to-use maps is available at the github page (https://github.com/Trajetorias-Sinbiose/map_repository). Detailed metadata tables should be consulted for further information regarding the calculations of the indicators. Users of the dataset should be aware of its limitations, as described in the technical validation section. Any future improvement will be reported on the project’s github site.

## Supplementary information

Supplementary Information

## Data Availability

Health and demographic data harmonization scripts, coded in R, are available at https://github.com/Trajetorias-Sinbiose/Trajetorias_dataset. Please refer to the readme file for further instructions. Information on the script developed to produce the MPI-Trajetorias indicators is also found there.
