# Supplementary material for: Trajetorias: a dataset of environmental, epidemiological, and economic indicators for the Brazilian Amazon
Source: Sci Data. 2023 Feb 2;10:65. doi: 10.1038/s41597-023-01962-1 (PMC9895449; doi:10.1038/s41597-023-01962-1)
Supplement: Supplementary file 1 — Supplementary Information [file 41597_2023_1962_MOESM1_ESM.pdf]

Supplementary material of the manuscript “Trajetorias: a dataset of environmental, epidemiological, and economic indicators for the Brazilian Amazon”

Rorato et al.

Manuscript Number: SDATA-22-01187

This supplement contains tables and figures that further describe the Trajetorias dataset.

Table of contents

Environmental dimension

Figure S1. Average minimum temperature per month in the five climatological regions of the Amazon. Regions 1 and 2 (east side) present a linear increase in temperature through the years.

Figure S2. Linear correlation between all environmental variables in the dataset in the first period of observation (reference 2006).

Figure S3. Linear correlation between all environmental variables in the dataset in the second period of observation (reference 2017).

Epidemiological dimension

Table S1. Summary of main epidemiological events in the Amazon region in the two observation periods, 2004-2008 and 2015-2019.

Figure S4. Linear correlation between epidemiological indices in rural and urban areas in the first period (2004-2008) and the second period (2015-2019) of observation. Linear correlation between epidemiological indices in total areas (rural + urban) in the first period (2004-2008) and the second period (2015-2019) of observation.

Economic dimension

Table S2. Variables and weights used for the computation of the rural and urban multidimensional poverty indices.

Figure S5. Linear correlation between economic indices in rural and urban areas in the first period (2000) and the second period (2010) of observation.

Environmental dimension

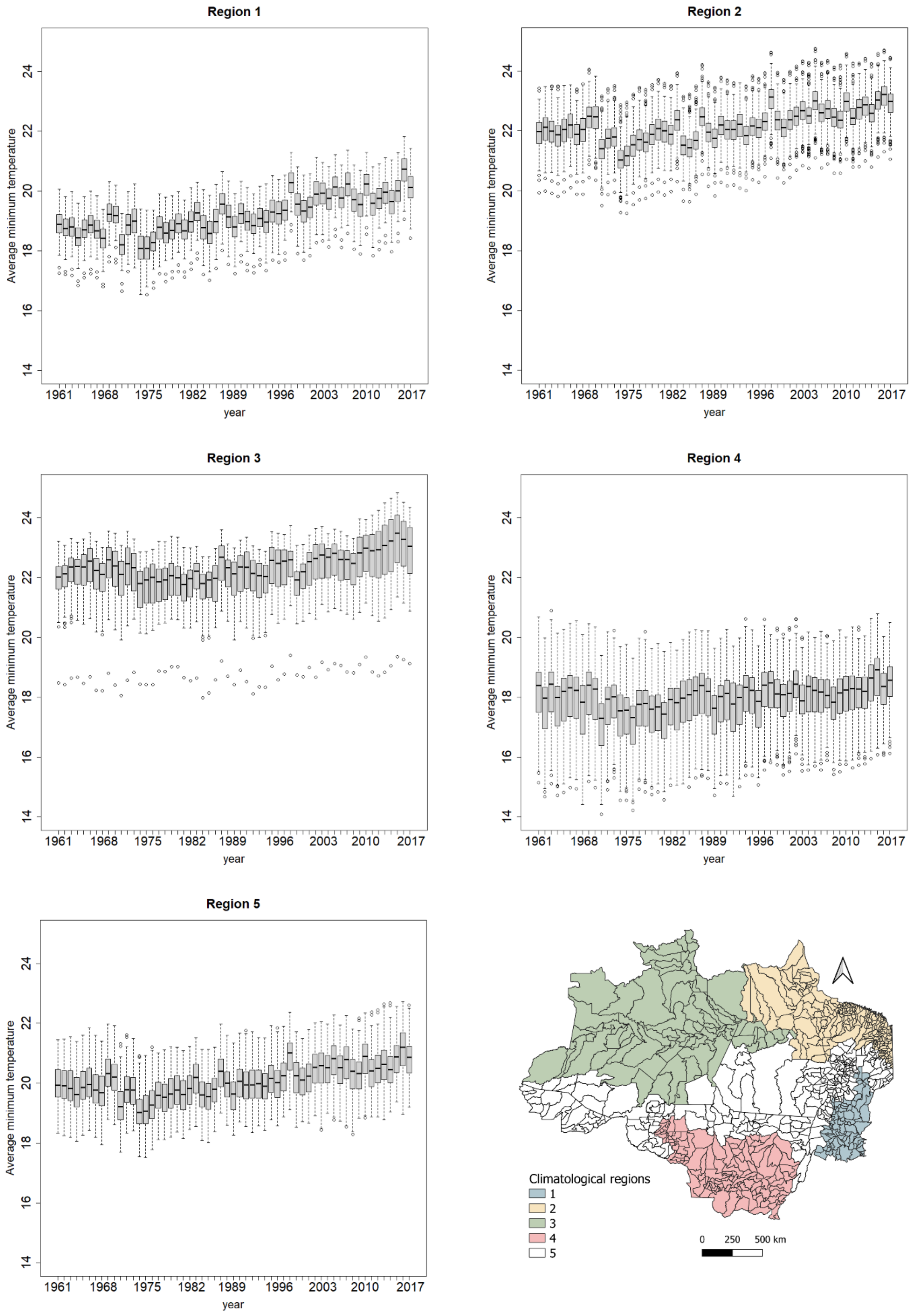

Figure S1. Average minimum temperature per month in the five climatological regions of the Amazon. Regions 1 and 2 (east side) present a linear increase in temperature through the years.

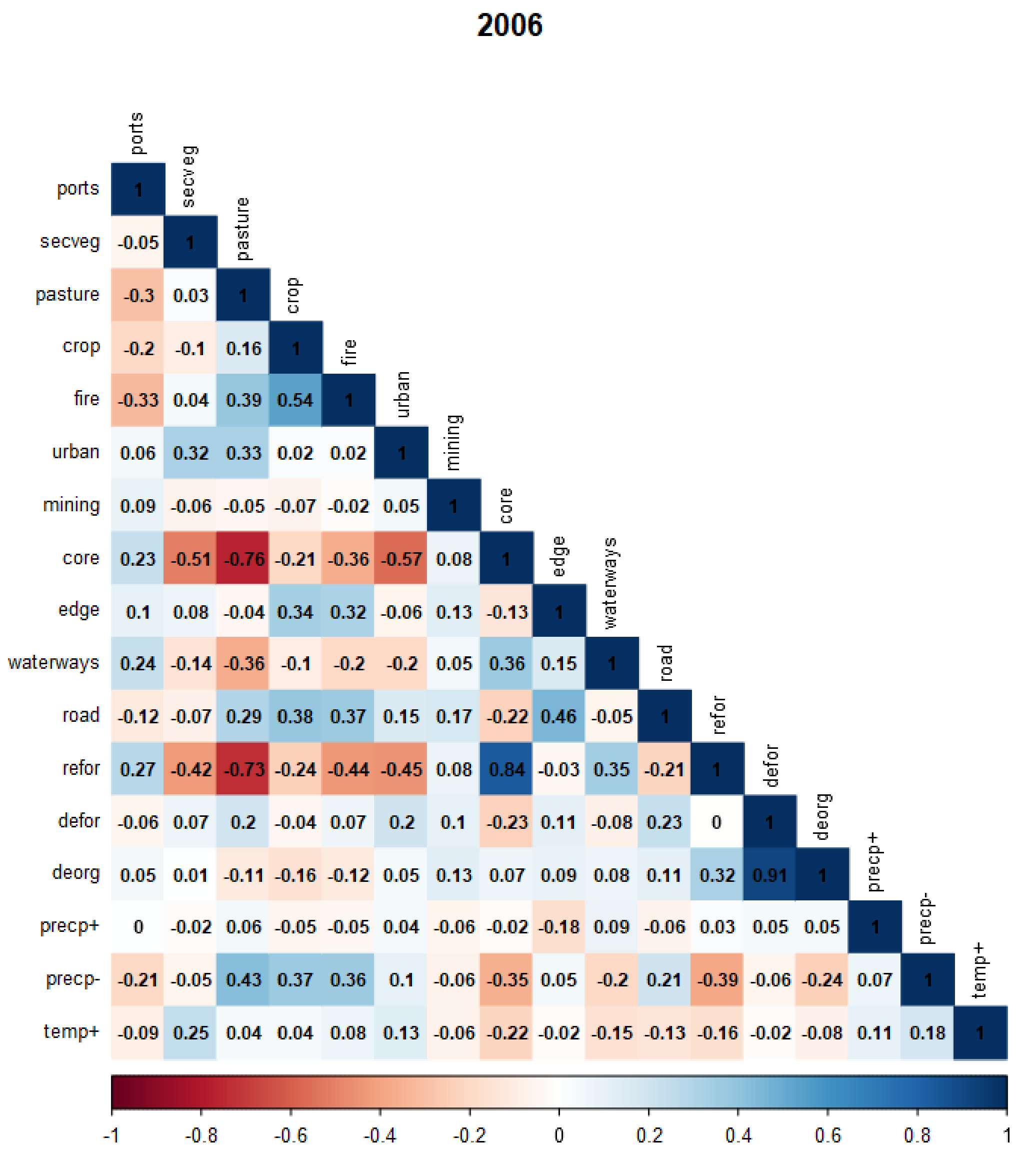

Figure S2. Linear correlation between all environmental variables in the dataset in the first period of observation (reference 2006).

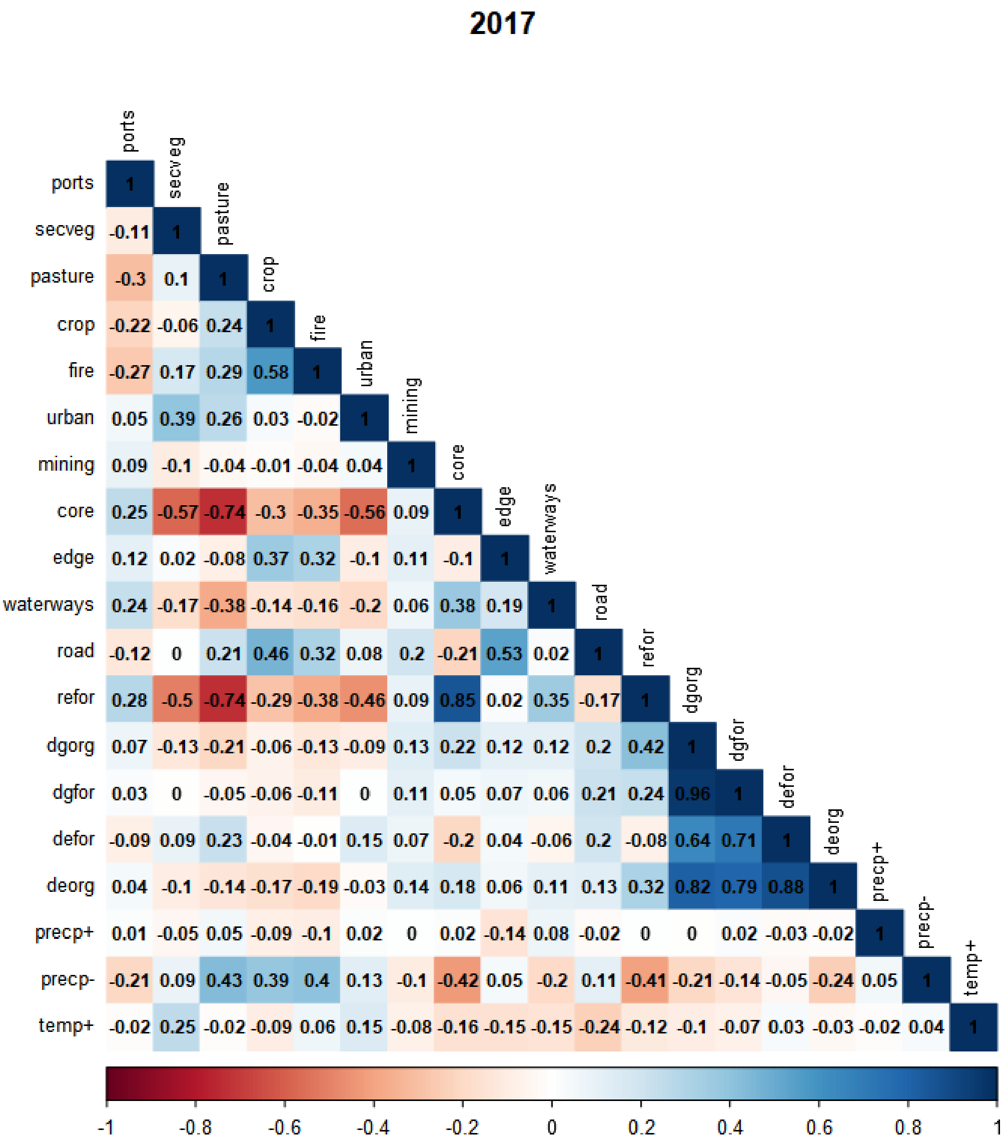

Figure S3. Linear correlation between all environmental variables in the dataset in the second period of observation (reference 2017).

Epidemiological dimension

Table S1. Summary of main epidemiological events in the Amazon region in the two observation periods, 2004-2008 and 2015-2019.

Disease	Period	epidemic period?	years with epidemics	description
Dengue	2004-2008	Yes	2008: MT, TO, RO, RR	DENV-2 was the dominant virus
	2015-2019	Yes	2015: AC, AM, MT, TO<br>2016: AC, MT, RO, TO	Introduction of Chikungunya (2015) and Zika (2016) possibly affecting dengue detection
Malaria	2004-2008	Yes	2004: RO,RR<br>2005: AC, RO, RR<br>2006: AC, AM, RO<br>2007: AC, AM, RO	Mainly *P. vivax* . Epidemic years with annual parasite index > 50.
	2015-2019	No	-	-
Malaria (*P. falciparum*)	2004-2008	Yes	2004: AM<br>2005: AC, AM, AP, RO, RR<br>2006: AC, AM, AP, RO, RR<br>2007: AC, RO	Epidemic years with annual parasite index > 10
	2015-2019	-	-	-
Chagas disease	2004-2008	Yes	2004: AM, AP, MA, RO<br>2005: RO<br>2006: PA, MT<br>2007: AM, PA<br>2008: AP, MA, TO	these are years with above average incidence
	2015-2019	Yes	2015: RR<br>2016: AC, PA<br>2017: AP, AM, MT<br>2018: AP, MA, PA, RO, RR, TO<br>2019: AC, AP, AM, MT, RO, RR, TO	these are years with above average incidence
Visceral leishmaniasis	2004-2008	Yes	2004: PA, RO, RR<br>2005: RR<br>2006: PA<br>2007: TO<br>2008: TO, MT	these are years with above average incidence
	2015-2019	Yes	2016: RR, TO<br>2017: MT, PA, RR<br>2018: PA, TO	these are years with above average incidence

Disease	Period	epidemic period?	years with epidemics	description
Cutaneous Leishmaniose	2004-2008	Sim	2004:AC, AP, AM, MT,PA, RO, TO<br>2005:AC, AM, MT, PA, RO, RR, TO<br>2006: MT, RR, TO<br>2007: AM, RR<br>2008: RR	these are years with above average incidence
	2015-2019	Sim	2015: AC,AM,MT,PA,RO,RR,TO<br>2016:AP<br>2017:AC,AP,AM,MT,PA,RO<br>2018:AP,AM,RO,RR<br>2019:PA,TO	these are years with above average incidence

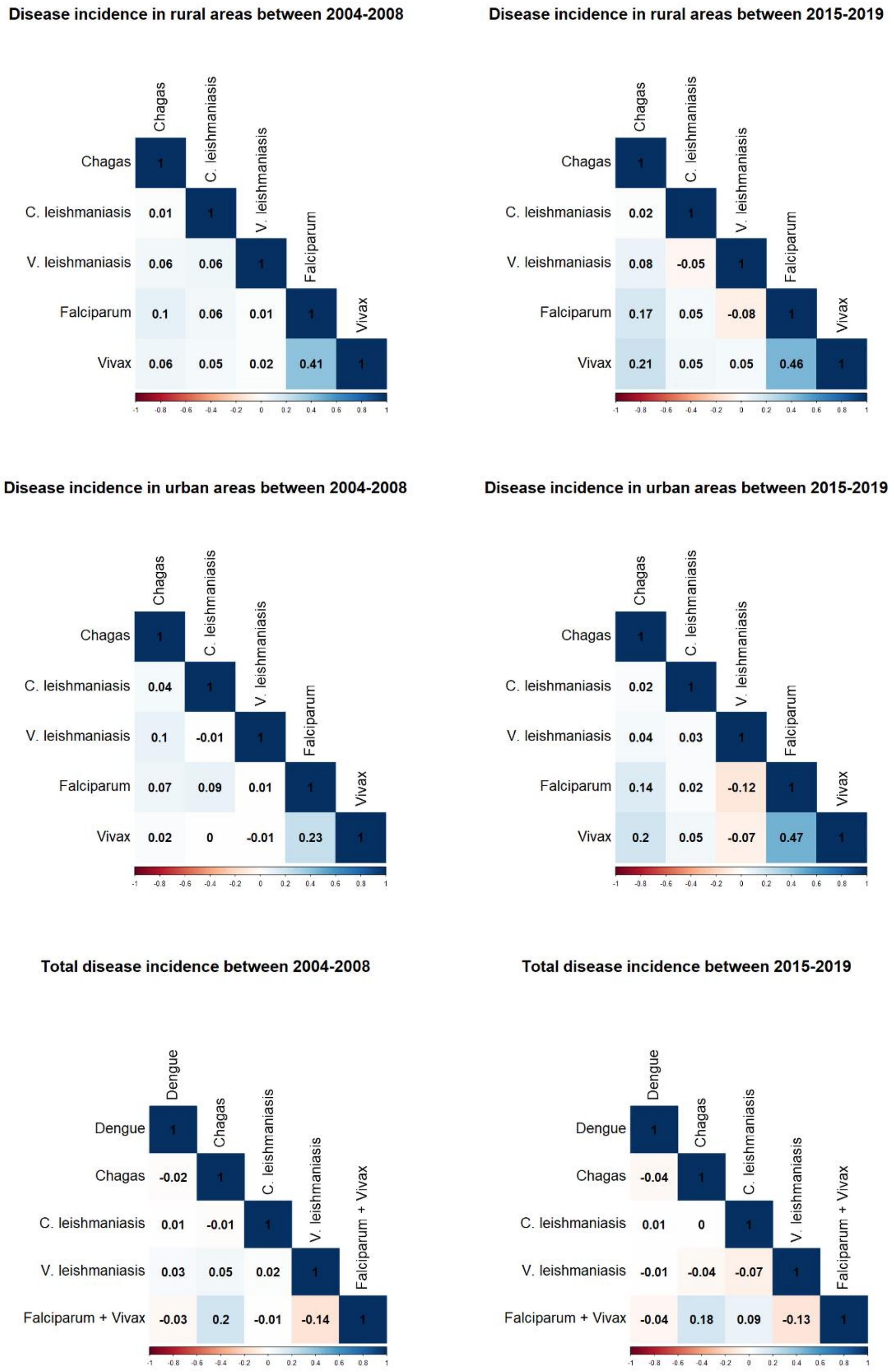

Figure S4. Linear correlation between epidemiological indices in rural and urban areas in the first period (2004-2008) and the second period (2015-2019) of observation. Linear correlation between epidemiological indices in total areas (rural + urban) in the first period (2004-2008) and the second period (2015-2019) of observation.

Economic dimension

Table S2. Variables and weights used for the computation of the rural and urban multidimensional poverty indices.

Dimension	Description		Weight			
			Urban PMI		Rural PMI	
			2000	2010	2000	2010
Education (¼)	schooling of adults	at least one household > 18 years of age, excluding the head of household, who did not complete elementary school	1/12	1/12	1/12	1/12
	schooling of head of household	head of the household is illiterate	1/12	1/12	1/12	1/12
	schooling of children	at least one household, aging between 4 and 14, not in the school	1/12	1/12	1/12	1/12
Health (¼)	death of newborn	at least one death of newborn in the household	1/4	1/12	1/4	1/12
	death of infants	at least one death of infants between 1 and 5 years of age	-	1/12	-	1/12
	early death	at least one death of householders between 6 and 73 years of age (life expectancy)	-	1/12	-	1/12
Living conditions: Housing (¼)	house constructi on quality	not a permanent house made of timber or brick	-	1/28	-	1/32
	temporary or collective house	temporary, collective or improvised house (p.e., tent)	1/24	1/28	1/28	1/32
	house size	(# householders / # dorms) > 5	1/24	1/28	1/28	1/32
	sewage	house not linked to a sewage network or septic tank)	1/24	1/28	1/28	1/32

	garbage	house without garbage collection services	1/24	1/28	1/28	1/32
	energy	house without electricity	1/24	1/28	1/28	1/32
	water	house without water services, or well/spring in the property, or do not have piped water.	1/24	1/28	1/28	1/32
	land ownership	permanent house in ceded land or other similar condition	-	-	1/28	1/32
Living conditions: income and consumer goods (¼)	social security	the only source of income is social security or unemployment aid, or student aid, or family allowance or other social programs.	1/20	1/20	1/20	1/20
	paid labor	head of the household without payment from main job in the last month	1/20	1/20	1/20	1/20
	food storage	household without a fridge	1/20	1/20	1/20	1/20
	communication	household without computer or cell phone, or TV or radio	1/20	-	1/20	-
		household without computer or cell phone with internet access, or TV or radio	-	1/20	-	1/20
	consumer goods	household without car for private use and washing machine.	1/20	-	-	-
		household without at least one of these items: car for private use, washing machine, phone.	-	1/20	-	-
	mobility	household without a car for private use.	-	-	1/20	-
		household without at least one of these items: car or motorcycle for private use.	-	-	-	1/20

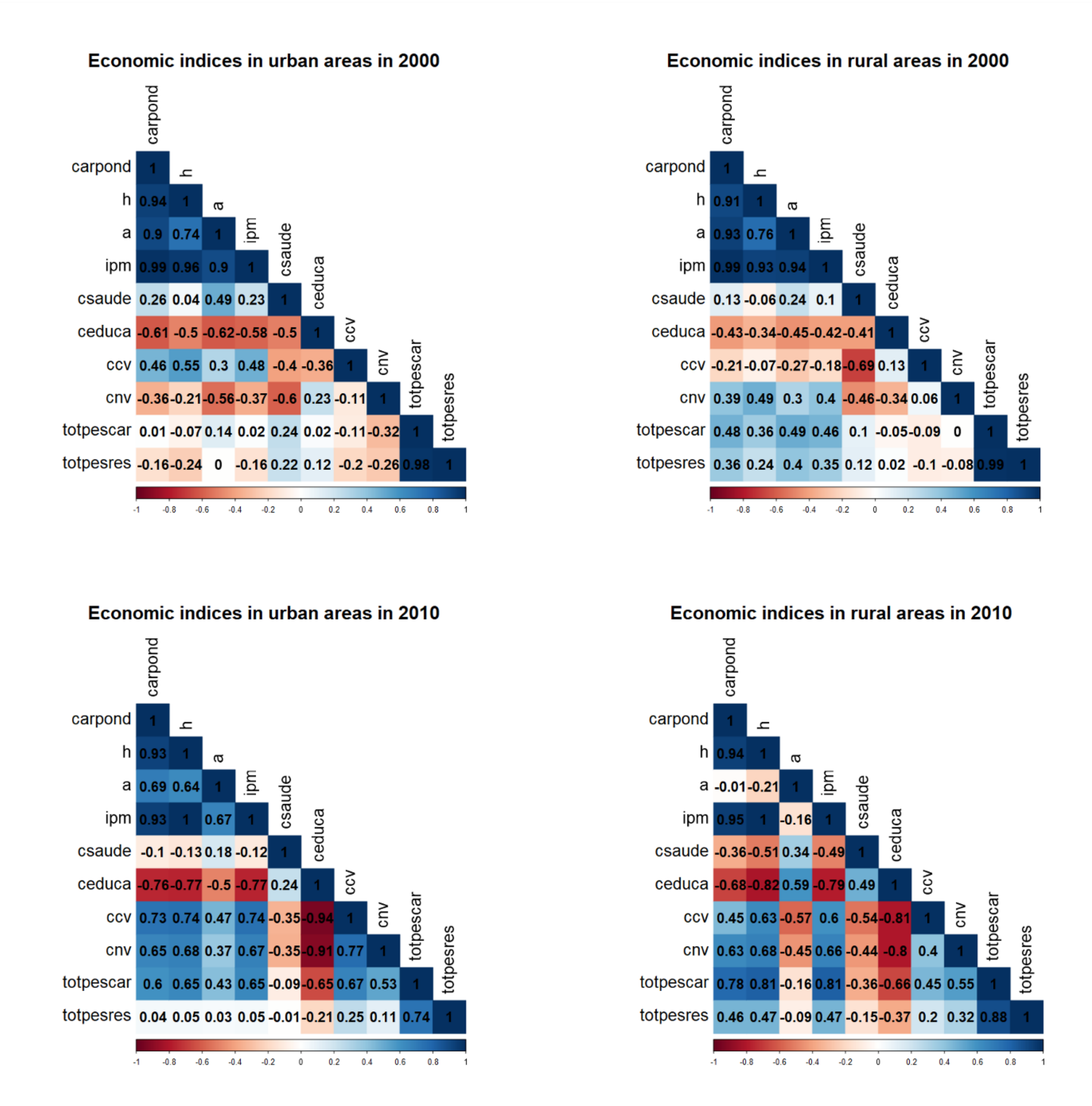

Figure S5. Linear correlation between economic indices in rural and urban areas in the first period (2000) and the second period (2010) of observation.
